# Green Synthesis of Copper Nano-Drug and Its Dental Application upon Periodontal Disease-Causing Microorganisms

**DOI:** 10.4014/jmb.2106.06008

**Published:** 2021-09-08

**Authors:** Sanaa M. F. Gad El-Rab, Sakeenabi Basha, Amal A. Ashour, Enas Tawfik Enan, Amal Ahmed Alyamani, Nayef H. Felemban

**Affiliations:** 1 Department of Biotechnology, Faculty of Science, Taif University, P.O. Box 888, Taif 21974, KSA; 2 Department of Botany and Microbiology, Faculty of Science, Assiut University, Assiut 71516, Egypt; 3 Department of Preventive and Community Dentistry, Faculty of Dentistry, Taif University, Taif 26571, Saudi Arabia; 4 Department of Oral and Maxillofacial Surgery and Diagnostic Sciences, Oral Pathology Division, Faculty of Dentistry, Taif University, Taif 21431, Saudi Arabia; 5 Dental Biomaterials, Faculty of Dentistry, Taif University, Taif 26571, Saudi Arabia; 6 Dental Biomaterials, Faculty of Dentistry, Mansoura University, Dakahleya 35516, Egypt; 7 Preventive dentistry department, Faculty of Dentistry, Taif University, Taif 26571, Saudi Arabia

**Keywords:** Antibiotic, antimicrobial activity, copper nanoparticles, periodontal disease

## Abstract

Dental pathogens lead to chronic diseases like periodontitis, which causes loss of teeth. Here, we examined the plausible antibacterial efficacy of copper nanoparticles (CuNPs) synthesized using *Cupressus macrocarpa* extract (CME) against periodontitis-causing bacteria. The antimicrobial properties of CME-CuNPs were then assessed against oral microbes (*M. luteus*. *B. subtilis*, *P. aerioginosa*) that cause periodontal disease and were identified using morphological/ biochemical analysis, and 16S-rRNA techniques. The CME-CuNPs were characterized, and accordingly, the peak found at 577 nm using UV–Vis spectrometer showed the formation of stable CME-CuNPs. Also, the results revealed the formation of spherical and oblong monodispersed CME-CuNPs with sizes ranged from 11.3 to 22.4 nm. The FTIR analysis suggested that the CME contains reducing agents that consequently had a role in Cu reduction and CME-CuNP formation. Furthermore, the CME-CuNPs exhibited potent antimicrobial efficacy against different isolates which was superior to the reported values in literature. The antibacterial efficacy of CME-CuNPs on oral bacteria was compared to the synergistic solution of clindamycin with CME-CuNPs. The solution exhibited a superior capacity to prevent bacterial growth. Minimum inhibitory concentration (MIC), minimum bactericidal concentration (MBC), and fractional inhibitory concentration (FIC) of CME-CuNPs with clindamycin recorded against the selected periodontal disease-causing microorganisms were observed between the range of 2.6–3.6 μg/ml, 4-5 μg/ml and 0.312-0.5, respectively. Finally, the synergistic antimicrobial efficacy exhibited by CME-CuNPs with clindamycin against the tested strains could be useful for the future development of more effective treatments to control dental diseases.

## Introduction

As of 2017, it was estimated that about 3.5 billion people have dental diseases such as tooth caries and periodontitis infections in their permanent teeth [1]. Periodontal disease is one of the major public health problems in many countries [2, 3]. It presents as a chronic, inflammatory, and infectious disease that affects the teeth and surrounding structures [4, 5], causing in the most severe cases, excessive gingival bleeding and inflammation, increased dental mobility, and finally, tooth loss [6]. Studies have shown that disturbance of microbiota may activate *P. aeruginosa*, *E. coli*, *S. pyogenes*, and *B. cereus* [7-9]. In the periodontal site of chronic patients, high distribution of *B. cereus*, *S. pyogenes*, *P. aeruginosa*, and *E. coli* commonly occurs [10]. Research has also suggested periodontal disease as the most common oral infectious disease that is predominated by microorganisms [11]. Also, bacterial resistance in biofilms to anti-microbial treatment can be up to 1,000 times greater than that of planktonic organisms [12] which presents a major global health problem [13] and causes tooth loss [14, 15]. Clindamycin is an antibiotic that is usually advised as effective therapy because of its excellent coverage of typical oral flora [16]. Currently, microbial resistance to conventional antibiotics is considered an important health problem and has raised the demand for more effective solutions [17]. The discovery of novel, strategic and effective antimicrobial agents to prevent microbial infections is needed [18] to prevent plaque formation and maturation as the first line of therapy [19]. The study of synergistic effects of metal nanoparticles with antibiotics and their antimicrobial effect on periodontal disease-causing microorganisms is an important aim.

Copper nanoparticles (CuNPs) exhibit high antimicrobial activity against different species of microorganisms including fungi, gram-negative and gram-positive bacteria; however, there have been no reports on their activity against dental pathogens. In antimicrobial therapies, the use of CuNPs would require and depend on therapeutic time and concentration [20]. Eco-friendly materials such as microbes, plants, and enzymes are used for the biosynthesis of CuNPs because they are nontoxic materials as well as being simple to use and low cost [21-23].

In this study, we adopted dental prophylaxis as our objective and sought to synthesize antibacterial nano copper using an herbal formulation of CME as a reducing agent against periodontal infectious disease-causing microorganisms such as gram-positive *B. subtilis*, *M. luteus*, in addition to gram-negative *P. aeruginosa*. We also evaluated the antimicrobial efficiency of combining antibiotics and biosynthesized CuNPs to overcome antibiotic resistance.

## Materials and Methods

### Preparation of *Cupressus macrocarpa* Aqueous Extract

*Cupressus macrocarpa* plant was identified by Prof. Ahmed A. El-Settawy (Head of Forestry and Wood Technology Department at the Faculty of Agriculture, Alexandria University) and given the voucher number Zidan313 [24]. Aqueous extract of *Cupressus macrocarpa* leaves was prepared by taking 25 g of leaves, rinsing it in a glass beaker containing deionized water (100 ml), and heating at 85°C for 30 min and then filtering and storing it for subsequent use [25].

### Biosynthesis of CuNPs

Forty milliliters of copper acetate (Sigma-Aldrich, USA) and then 10 ml of CME were added in an Erlenmeyer flask. Then, 10 ml of the CME was added. The mixture was irradiated in a microwave oven (Matsushita Electric Industrial Co., Ltd. Panasonic, Japan) at 800 W for 1 h until the formation of copper nanoparticles (CME-CuNPs), and allowed to cool at room temperature [26].

### Characterization of CME-CuNPs

Characterization of CME-CuNPs was done using the following methods:

*UV-Visible spectrum*: UV–vis spectrometer (Shimadzu UV-1650) was used to record absorbance in the range of 300–700 nm and monitor the rate of CME-CuNP formation [27].

*Transmission electron microscopy (TEM)*: TEM analysis was done using the TEM JEOL 100 kV (Assiut Electron Microscope Unit). The CME-CuNPs were prepared by placing a drop of the suspension on carbon-coated copper grids and allowing it to dry on the grid for 4 min. The shape and size of CME-CuNPs were determined from the TEM micrograph [28].

### X-Ray Diffraction Analysis

The nature and size of the CME-CuNPs were determined using Shimadzu XRD (Shimadzu XD-3A, Japan). The nanoparticle size was calculated using the formula of Debye–Scherrer [29].

*Fourier-transform infrared analysis (FTIR)*: The spectra of CME and CME-CuNPs were determined using a Shimadzu IR-470 Spectrometer (Shimadzu) in the range of 500–4,000 cm^-1^[30].

### Isolation and Identification of Bacterial Isolates

Ethical approval for the current study (Ref. No. 41- 1107-00152) was provided by the Ethics Committee of Research, Taif University, Taif, KSA. Bacterial isolates were obtained from the Faculty of Dentistry Medical Diagnosis and Infection Control Unit, Taif University, Taif, KSA. Isolation of bacteria collected from the mouths of patients (dental pulp, and dental plaque) was conducted by provisional researchers from patients infected with periodontal disease-causing microorganisms using sterile root canal instruments and the isolates were stored in sterile saline in a 10 ml saline kit. One hundred isolates were isolated on Mueller-Hinton agar (Thermo Scientific Oxoid), for 24 h at 37°C under aerobic condition and suspected single colonies were then identified by morphological/biochemical tests according to the criteria [31, 32].

### Molecular Characterization Using 16S rRNA Gene

Bacterial cells were used for collecting genomic DNA with the Wizard Genomic DNA Purification Kit (Promega, USA) and used as a PCR template. In 20 μl of the polymerase chain reaction (PCR) reaction solution containing forward primer 27F and reverse primer 1492R, one microliter of DNA template was added. Then, 35 amplification cycles of 16S rRNA genes were performed at 94°C for 45 s in the denaturation step, at 55°C for 60 s in the annealing step, and then at 72°C for 60 s in the extension step. DNA fragments were amplified up to 1,400 bp. The products of 16S rRNA of approximately 1,400 bp were sequenced using 518F/800R primer set. The sequencing of 16S rRNA has been analyzed on an automated Applied BioSystems, DNA sequencing system,(model 3730XL, USA). The similarity of bacterial strains was aligned using CLUSTAL W (1.81) phylogenetic tree obtained from the nucleotide sequence databases.

### Antibacterial Activity of CME-CuNPs

Micro-Dilution Test for Determination of MIC and MBC

The MBC of CME-CuNPs, clindamycin, or both is defined as the lowest concentration of an antimicrobial agent killing the majority (99.99%) of bacterial inoculums. Since the MIC of CME-CuNPs, clindamycin, or both relates to their inhibitory ability, it is possible that if the antimicrobial agent were removed, the bacteria would begin to grow again [33]. The test was performed with serial dilutions of clindamycin (from 64 μg/ml to 8 μg/ml), CME-CuNPs (from 27 μg/ml to 9 μg/ml) or clindamycin with CME-CuNPs (from 5 μg/ml to 2.6 μg/ml) arranged across the rows in Mueller-Hinton broth and inoculation of the wells of a micro-dilution plate with the bacterial culture. The MIC and MBC of CME-CuNPs, clindamycin, or both were determined against the tested isolates [34]. All samples of clindamycin, CME-CuNPs, or CME-CuNPs with clindamycin (1 μg/ml:1 μg/ml) were tested in triplicate, and the test was repeated five separate times.

### Antibacterial Synergy Test

The antibacterial synergy in vitro test was carried out using a checkerboard synergy method based on the value of the FIC (fractional inhibitory concentration). The FIC value was calculated by comparing the MIC of each agent CME-CuNP and clindamycin with the MIC of the CME-CuNP and clindamycin combination. Clindamycin was added in concentrations between 64 and 8 μg/ml and CME-CuNPs in a range of 10–30 μg/ml. These effects can be quantified by the application of mathematical expressions: the fractional inhibitory concentration (FIC) of combinations among CME-CuNPs and clindamycin against tested strains. For two antibacterial agents, CME-CuNPs (A) and clindamycin (B) acting individually or in combination:

FIC_A_=MIC (_A in the combination of B_)/MIC (_A alone_)

FIC_B_=MIC (_B in the combination of A_)/MIC (_B alone_)

FIC= FIC_A_ + FIC_B_

An FIC index of < 0.5 indicates synergism, > 0.5–1 indicates additive effects, > 1 to < 2 indifference, and ≥ 2 is considered to be antagonism [34].

### Statistical Analysis

Descriptive summary statistics were obtained for all independent and outcome variables. The mean difference was tested using analysis of variance (ANOVA) followed by Tukey's post hoc and *t*-test. The analysis was obtained using the Statistical Package for Social Science version 17 (SPSS INC Chicago link). All statistical tests were two-sided, and the significance level was set at *p* < 0.05.

## Results

### Biosynthesis of CME-CuNPs

In the current research, CME-CuNPs were biosynthesized by reducing copper acetate to CME-CuNPs using CME. The formation of CME-CuNPs in the reaction mixture is indicated by a change in the color from blue to brown.

### Characterization of CME-CuNPs

**Fig. 1:**
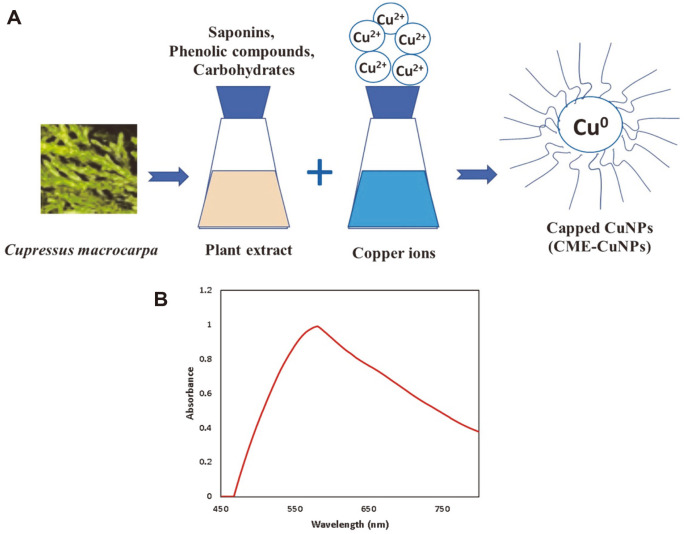
(**A**) Illustration showing biosynthesis of CME-CuNPs. Notes: Photograph showing the formation of CME-CuNPs using *Cupressus macrocarpa* extract. (**B**) UV–visible spectrum of CME-CuNPs.

UV–Vis spectroscopy of CME-CuNPs: UV–Vis absorption was studied after the formation of CME-CuNPs was dispersed and absorbance was measured by using a UV–visible spectrophotometer between 450 and 800 nm. CME-CuNPs showed peaks at 577 nm as shown in Fig. 1. The UV–visible peak at 577 nm was for pure CME-CuNPs.

### Transmission Electron Microscope Analysis

**Fig. 2:**
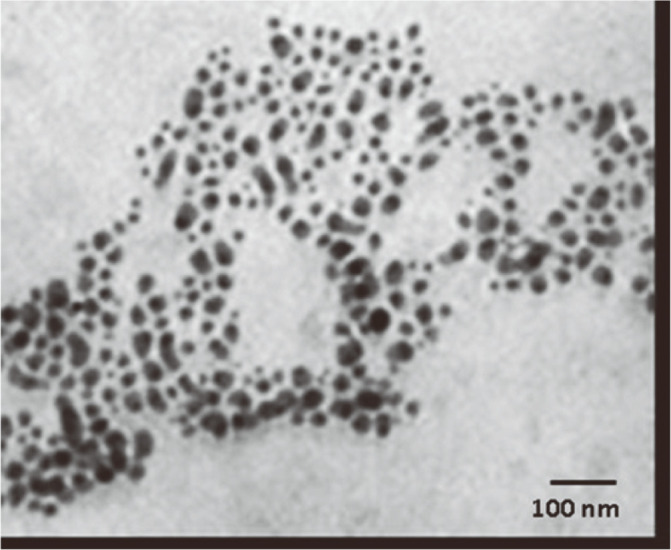
TEM images of the synthesized CME-CuNPs.

The CME-CuNPs were characterized by TEM analysis (Fig. 2). The average size of the CME-CuNPs was estimated to be at 11.3-22.4 nm, via TEM analysis. TEM analysis of the CME-CuNPs showed spherical to oblong, polydispersed shapes (Fig. 2).

### X-Ray Diffraction (XRD)

**Fig. 3:**
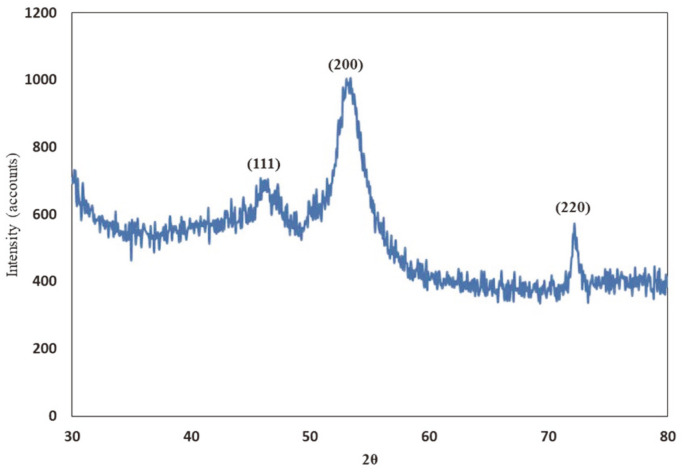
XRD patterns of the synthesized CME-CuNPs with aqueous extract of *Cupressus macrocarpa*.

X-ray diffraction (XRD) pattern showed four major peaks at 2θ values of 47°, 54°, and 72°. These characteristic peaks could be attributed to reflection planes (111), (200), and (220) of the face-centered cubic crystalline (FCC) structure of pure metallic copper (Fig. 3).

### Comparison of FTIR Spectra of *Cupressus macrocarpa* Extract, CME-CuNPs

**Fig. 4:**
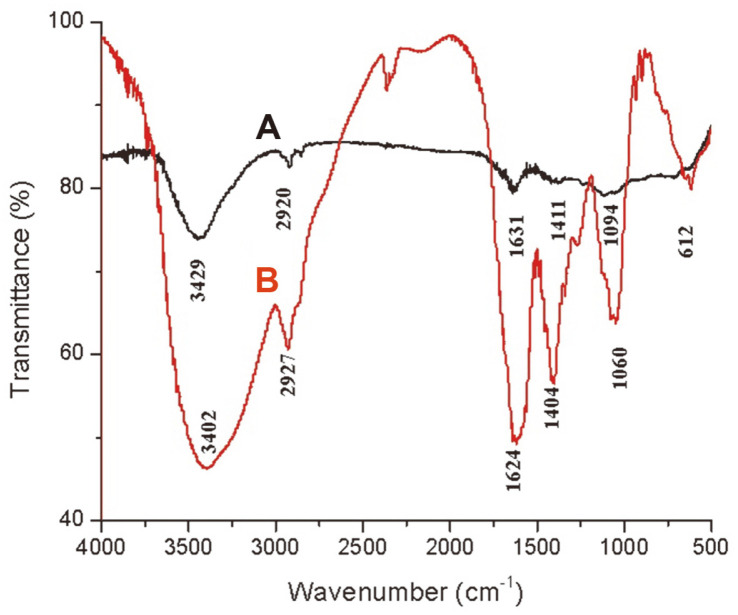
FTIR spectra of *Cupressus macrocarpa* extract (**A**) and the synthesized CME-CuNPs (**B**).

Fourier-transform infrared analysis (FTIR) was used to characterize the CME and the resulting CME-CuNPs. Absorbance bands were observed in the region 500 to 4,000 cm^-1^, (Figs. 4A and 4B). Fig. 4A showed the absorption bands of plant extract. There is a broad and strong band in the region 3,429 cm−1 ascribed to O–H stretching vibrations. According to a known standard, a band in the 2,920 cm−1 region originates from C–H (hydrocarbon) stretching vibration, 1,631 cm^-1^, denoting an amine group related to proteins, 1,411 cm^-1^, representing methylene -CH bending bond, and 1,094 cm^-1^ of primary alcohol/C-O- stretching. The CME caused a reduction of copper ions. Fig. 4B showed the absorption bands of CME-CuNPs. Peaks at 3,402, 2,927, 1,631, 1,404, and 1,060 cm^-1^ represent the OH functional groups, an amine group (NH), stretching -CH, and C-O stretching vibrations, respectively. Polyphenolics, proteins, amino acids, and carbohydrate compounds were adsorbed on the surface of the CME-CuNPs.

### Isolation and Identification of Drug-Resistant Bacteria

One hundred isolates were collected on Mueller-Hinton agar at 37°C. Then, the suspected single colonies were identified as *M. luteus* (50%), *B. subtilis* (12.5%), and *P. aeruginosa* (12.5%) by morphological/biochemical tests according to the criteria.

The strains were morphologically and biochemically characterized as *M. luteus*, *B. subtilis*, and *P. aeruginosa* and designated as *M. luteus* MIC1, *B. subtilis* BAC1, and *P. aeruginosa* PSE1, respectively. The aerobic strains MIC1, ABC1, and PSE5 were chosen for the current study (Table 1).

### 16S rRNA Analysis

Partial 16S rRNA gene sequences were used to identify multidrug-resistant bacterial isolates. MIC1, BAC1, and PSE5 were carried and sequenced. The 16S rRNA gene sequences of the bacterial isolates from the dental pulp and plaque were deposited in the DDBJ/EMBL/GenBank nucleotide sequence databases with the following accession numbers (Table 2): MIC1 strain (LC628029), BAC1 strain (LC628030), and PSE5 strain (LC628031). The 16S rRNA gene sequences of strains were found to be 1310, 1240, and 1312 nt in length, for strain MIC1, BAC1, and PSE5, respectively.

A dendrogram demonstrating the results of the 16S rRNA of *M. luteus* (MIC1), *B. subtilis* (BAC1), and *P. aeruginosa* (PSE5) analysis is displayed in Figs. 5A, 5B, 5C. The results show the highest similarity of isolates MIC1, BAC1, and PSE5 to members of the Micrococcus, Bacillus, and Pseudomonas group, respectively. Moreover, the 16S rRNA sequences of the isolates MIC1, BAC1, and PSE5 are the most closely associated with *M. luteus*, *B. subtilis*, and *P. aeruginosa* (Table 2). The 16S rRNA gene of MIC1, BAC1, and PSE5 displayed 98.31%, 96.43%, and 97.22 similarities to *Micrococcus luteus* DSM 20030 (NR037113.1), *Bacillus subtilis* subsp. *subtilis* 168 (NR102783.2), and *Pseudomonas aeruginosa* ATCC 10145 (NR114471.1), respectively.

### Antimicrobial Activity

Minimum Inhibitory Concentration (MIC) and Minimum Bactericidal Concentration (MBC) Assays

In this study, three strains resistant to clindamycin were used. The MIC of CME-CuNPs was found to be 13.5, 18, and 18 μg/ml against *M. luteus*, *B. subtilis*, and *P. aeruginosa*, respectively (Table 3), while the MIC of CME-CuNPs with clindamycin was found to be 2.6, 3.6, and 3.6 μg/ml, respectively. Moreover, the MBC of CME-CuNPs was determined to be 20, 27, and 27 μg/ml for *M. luteus*, *B. subtilis*, and *P. aeruginosa*, respectively, while the MBC of CME-CuNPs with clindamycin was determined to be 4, 5, and 5 μg/ml, respectively. The MICs of CME-CuNPs with clindamycin significantly decreased by more than 5 folds compared to CME-CuNPs alone while it decreased by more than 3-10 folds compared to clindamycin alone. Our data showed that CME-CuNPs have a small size with high antimicrobial activity. As shown in Table 4, MICs of CME-CuNPs were the lowest concentration (13.5–18 μg/ml) compared to MICs of CuNPs in earlier studies (40 mg/ml, 75 μg/ml, 100 μg/ml, and 150-225 μg/ml) on antibacterial activity against bacteria.

### Antibacterial Synergy Test

The FIC of CME-CuNPs and the clindamycin combination were investigated and are summarized in Table 5. This experiment showed that CME-CuNPs acted synergistically (*p* < 0.5) with clindamycin against *B. subtilis*, *M. luteus*, in addition to *P. aeruginosa*.

### Mechanism of Action

**Fig. 6:**
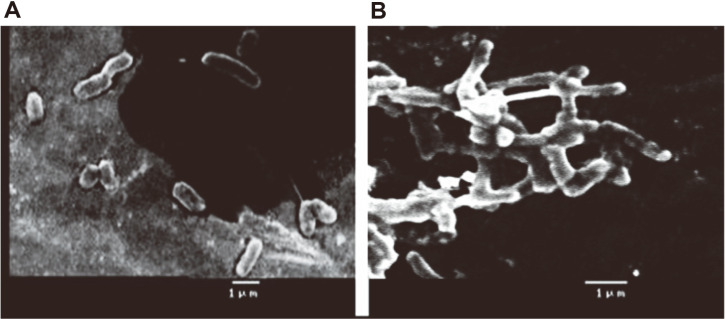
SEM observation of control *B. subtilis* (a) and images shows the CME-CuNP treated *B. subtilis* (b) showing membrane damage, and cell elongation in treated cells.

The CME-CuNP treated *B. subtilis* was observed with distorted membrane morphology, and cell elongation,(Fig. 6B) while the untreated control *B. subtilis* cells were found to be compact, tightly packed cells (Fig. 6A). In our results, the bacteria cells were distorted and CME-CuNPs act as an inhibitor for selected bacteria.

## Discussion

In this study, we applied a combination of CME-CuNPs and clindamycin. When CME-CuNPs with clindamycin antibacterial properties were compared with CME-CuNPs alone against tested bacteria, CME-CuNPs with clindamycin proved to be stronger. Based on our results, the present study confirmed the success of the biosynthesis of CME-CuNPs from CME. The CME-CuNPs biosynthesis reaction progress was monitored for color change and UV-Vis spectrometric analysis. The change of color from blue to brown in the reaction mixture forming CME-CuNPs agreed with Wu *et al*. (2020) [35] who similarly observed the color change during the synthesis of CuNPs. The absorbance peaks recorded at 577 nm are specific to CME-CuNPs and were attributed to the surface plasmon resonance phenomenon which indicates the formation of CME-CuNPs. Various studies have also recorded similar CuNPs SPR peaks [26, 36, 37]. TEM images of nano copper: the appearance is spherical to oblong with a size of about 11.3-22.4 nm [38]. There are three lattice planes that are (111), (200), and (220) of the face-centered cubic (fcc) structure that was observed for CME-CuNPs at De Bragg’s reflection angles of 47°, 54°, and 72°, respectively. No other impurity peak was detected in the CME-CuNP sample. The XRD results reflected the purity of CME-CuNPs in the present study and this is in accordance with previous studies [39, 40].

The organic compounds in the CME reduced and stabilized the CME-CuNPs, thereby preventing agglomeration. The CME contains FTIR peaks at 3,402, 2,927, 1,631, 1,404, and 1,060 cm^-1^ representing the OH functional groups, an amine group (NH), stretching -CH, and C-O stretching vibrations, respectively. These functional groups of the CME related to polyphenolics, proteins, amino acids, and carbohydrate compounds were adsorbed on the surface of CME-CuNPs [41], and these components are functionalized in reducing and stabilizing the CME-CuNPs. Moreover, CME contains secondary metabolite components such as phenolics, flavonoids, saponins, tannins, and terpenes. For this reason, we used CME in the biosynthesis of CME-CuNPs. Also, CME has wound-healing, antibacterial, and anti-inflammatory properties [42].

Our results show that MIC1, BAC1, and PSE5 are compatible with the conclusions of the morphological and biochemical characterization. Various studies have demonstrated that *M. luteus*, *B. subtilis*, and *P. aeruginosa* can be isolated from dental plaque and dental pulp [43, 44].

The present study's antimicrobial results document the high potential of CME-CuNPs with clindamycin against periodontal disease-causing microorganisms (*M. luteus*, B subtilis, and *P. aeruginosa*) compared to CME-CuNPs or clindamycin alone. Our results demonstrated the greater synergistic effect of the solution containing CME-CuNPs and clindamycin and anti-periodontal disease agents against tested clinical strains, because of its low MIC values. The antibacterial efficacy of some nanoparticles such as CME-CuNPs have been evaluated in previous studies and different mechanisms have been proposed for their effects [45]. The antibacterial efficacy of CME-CuNPs was related to its capability to distort the bacterial cell and cause multiple effects of reactive oxygen species (ROS) formation, and liberation of Cu ions, which lead to lipid peroxidation, protein oxidation, and DNA degradation in bacterial cells [46]. The inhibition of growth in selected bacteria and ROS formation was caused by electrostatic attraction between negatively charged bacterial cells and positively charged nanoparticles [47].

Moreover, clindamycin has a synergy effect on the antibacterial efficacy of the CME-CuNPs on tested bacteria. The reaction between clindamycin and CME-CuNPs led to synergism. The clindamycin molecule contains active groups like hydroxyl and amide, which react easily with CME-CuNPs by chelation [48]. The rationale for using drug combinations (CME-CuNPs with clindamycin) is the expectation that effective combinations might lower the incidence of bacterial resistance, reduce the host toxicity of the antimicrobial agents or enhance bactericidal activity [44, 49].

In conclusion, the CME-CuNPs prepared by biological synthesis using CME are more secure, cost-effective, and eco-friendly. The biological synthesis approach for CME-CuNPs has many advantages, such as being simple to use with commercial viability. The synergistic solution of clindamycin with CME-CuNPs displayed more significant antimicrobial activity compared to clindamycin or CME-CuNPs alone against selected bacteria, such as *M. luteus*, *B. subtilis*, and *P. aeruginosa*. Therefore, the obtained eco-friendly biosynthesized CME-CuNPs can be used for their enhanced efficacy in bio-dental fields for exploiting the existing antibiotics and other drugs and will result in the development of cost-effective treatments for infections and periodontal diseases in the future.

## Figures and Tables

**Fig. 5A:**
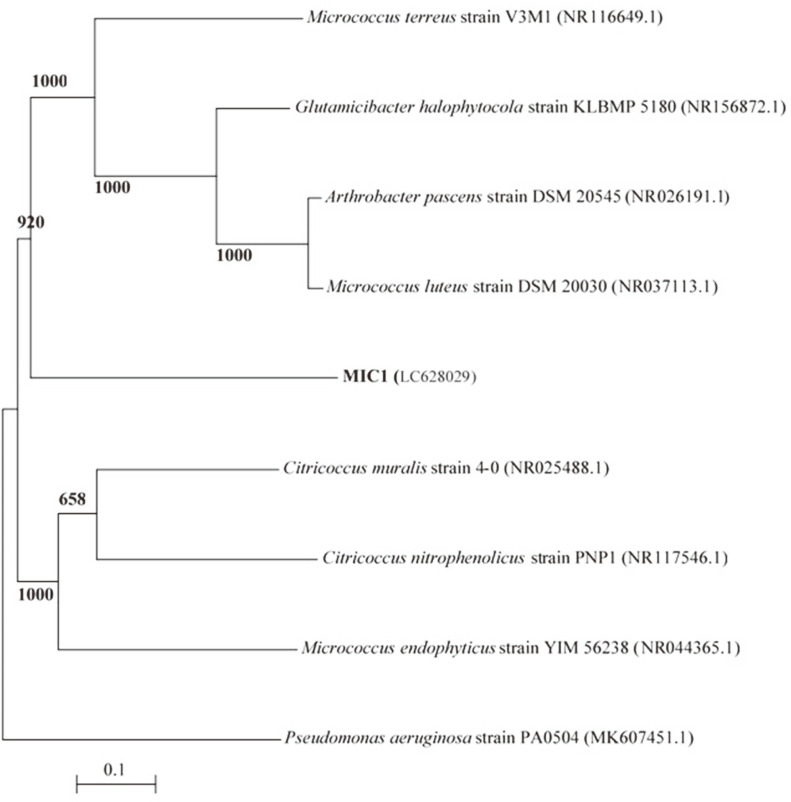
A phylogenetic tree of bacterial isolates relied on the nucleotide sequences of 16S rRNA genes, constructed by neighbor-joining method. The scale bar displays the genetic distance. The number presented next to each node displays the percentage bootstrap value of 1000 replicates. The *Pseudomonas aeruginosa* strain PA0504 was treated as the out-group. The GenBank accession numbers of the bacteria are presented in parentheses.

**Fig. 5B:**
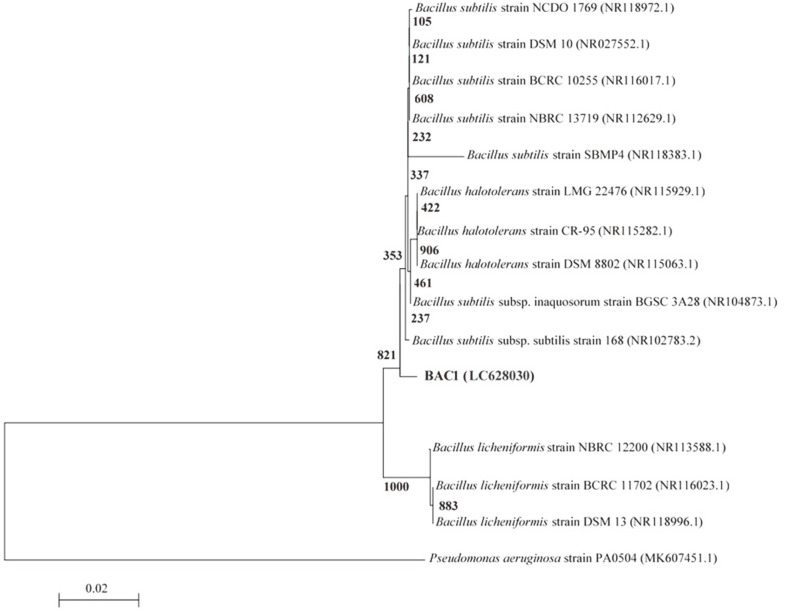
A phylogenetic tree of bacterial isolates relied on the nucleotide sequences of 16S rRNA genes, constructed by neighbor-joining method. The scale bar displays the genetic distance. The number presented next to each node displays the percentage bootstrap value of 1000 replicates. The *Pseudomonas aeruginosa* strain PA0504 was treated as the out-group. The GenBank accession numbers of the bacteria are presented in parentheses.

**Fig. 5C:**
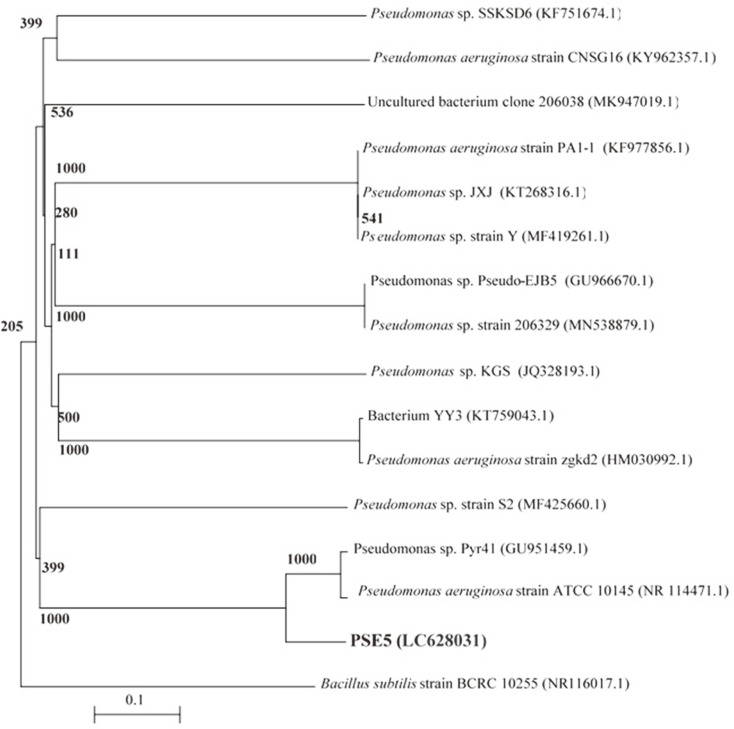
A phylogenetic tree of bacterial isolates relied on the nucleotide sequences of 16S rRNA genes, constructed by neighbor-joining method. The scale bar displays the genetic distance. The number presented next to each node displays the percentage bootstrap value of 1000 replicates. The *Bacillus subtilis* strain BCRC 10255 was treated as the out-group. The GenBank accession numbers of the bacteria are presented in parentheses.

**Table 1:** Morphological and biochemical characteristics of bacteria strains (MIC1, BAC1 and PSE5).

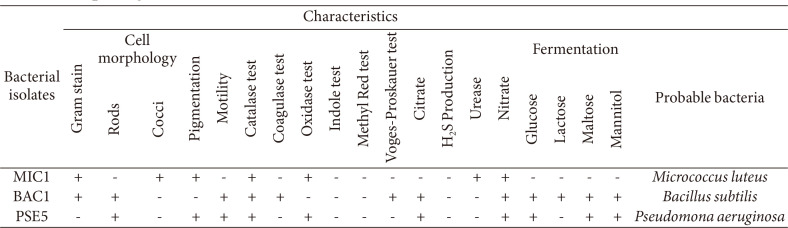

(-): Negative, (+): Positive

**Table 2:** The bacterial isolates (MIC1, BAC1 and PSE5) of oral infections.

Bacterial isolates	Accession No.	Closest neighbor
MIC1	LC628029	*M. luteus*
BAC1	LC628030	*B. subtilis*
PSE5	LC628031	*P. aeruginosa*

**Table 3:** MIC, MBC and of CME-CuNPs and CME-CuNPs with clindamycin (μg/ml) against oral bacteria in Mueller Hinton broth.

Isolate	Clindamycin (μg/ml)			CME-CuNPs (μg/ml)			CME-CuNPs with clindamycin (μg/ml)		

	MIC	MBC	MBC/MIC	MIC	MBC	MBC/MIC	MIC	MBC	MBC/MIC
*M. luteus* MIC1	8	16	2	13.5	20	1.5	2.6	4	1.5
*B. subtilis* BAC1	32	64	2	18	27	1.5	3.6	5	1.4
*P. aeruginosa* PSE1	32	64	2	18	27	1.5	3.6	5	1.4

MIC: Minimum inhibitory concentration, MBC: Minimum bactericidal concentration.

**Table 4:** Comparison of antibacterial activity for CME-CuNPs with earlier studies.

Materials	Size (nm)	Concentrations (μg/ml)	Bacteria	References
CuNPs	55 - 350 nm	40 mg/disc	*Staphylococcus aureus* (*S. aureus*), *Escherichia coli* (*E. coli*)	[50]
CuNPs	4.7 to 17.4 nm	100 μg/ml	*Bacillus*, *S. aureus*, *E. coli*, and *P. aeruginosa*	[51]
CuNPs	60–90 nm	75 μg/ml	*Streptococcus* sp. *E. coli*	[52]
CuNPs	131 nm	40 μg/ml	*Streptococcus mutans*	[53]
CuNPs	80 nm	150 -225 μg/ml	*Enterococcus faecalis*	[54]
CME-CuNPs	11.3-22.4 nm	13.5-18 μg/ml	*B. subtilis*, *M. luteus*, *P. aeruginosa*	Present study

**Table 5:** FIC index of combinations among CME-CuNPs and clindamycin against tested strains.

Bacterial isolates	FIC index	
MIC1	0.500	(S)
BAC1	0.312	(S)
PSE5	0.312	(S)

An FIC index of < 0.5 indicates synergism (S), > 0.5–1 indicates additive effects (AD), > 1 to < 2 indifference (ID), and ≥ 2 is considered to be antagonism (AN).
